# Assessment of Surface Treatment Degree of Steel Sheets in the Bonding Process

**DOI:** 10.3390/ma15155158

**Published:** 2022-07-25

**Authors:** Anna Rudawska, Izabela Miturska-Barańska, Elżbieta Doluk, Ewa Olewnik-Kruszkowska

**Affiliations:** 1 Faculty of Mechanical Engineering, Lublin University of Technology, Nadbystrzycka 36 St., 20-618 Lublin, Poland; i.miturska@pollub.pl (I.M.-B.); e.doluk@pollub.pl (E.D.); 2 Faculty of Chemistry, Nicolaus Copernicus University in Toruń, Gagarin 7 St., 87-200 Toruń, Poland; olewnik@umk.pl

**Keywords:** surface treatment, steel, adhesive joint, adhesive properties, surface roughness parameters, shear strength

## Abstract

The aim of the paper is to determine the influence of the surface treatment on the adhesive properties of steel sheet surfaces and the strength of the adhesive joints of steel sheets. The paper also aims to assess the degree of steel sheets’ surface treatment in the bonding process. Due to the many methods of surface treatment and types of materials, the assessment of the surface treatment method is extremely important in adhesive processes. Two variants of the surface treatment were used: without a paint coating and with a paint coating, divided into two groups (without degreasing and with degreasing). Additionally, in the case of the analysis of the steel samples without the paint coating, mechanical treatment was applied. Two-component epoxy adhesive, prepared on the basis of bisphenol A and a polyamide curing agent, was used to prepare the single-lap adhesive joints of the steel sheets. The tests determined: (i) the adhesive properties of the steel sheets’ surface based on the measurement of the contact angle of polar and apolar liquids (including wettability, work of adhesion, and surface free energy), (ii) surface roughness parameters (PN EN ISO 4287), and (iii) mechanical properties (load capacity and shear strength) of the steel sheets’ adhesive joints (EN DIN 1465). Contact angle measurements of the steel sheet surfaces showed that the polar liquid better reflects the obtained strength results of the analyzed adhesive joints than the apolar liquid. Furthermore, better wettability of the surface of steel sheets with both polar and apolar liquids was obtained for samples whose surface was subjected to degreasing. It can also be concluded that the wettability of the surface can be used as one of the indicators of the degree of the surface treatment for the bonding process.

## 1. Introduction

The bonding process consists of many operations that are performed in the correct sequence. The individual steps of bonding in the final stage affect the strength of the obtained adhesive joint. Surface treatment is the first step in achieving the strongest possible adhesive bonds in the adhesive joint [1,2,3,4,5]. Proper surface treatment allows an adhesive joint to be obtained with sufficiently high strength properties, which results in the proper operation of this joint and increases its resistance to operating factors [6,7,8,9].

Surface treatment is aimed at removing impurities and residues in the form of deposits, dust, grease, fats, oxides or microorganisms, increasing the surface roughness, which increases the adhesive properties of the joint and the ability to form interfacial bonds [8,10,11,12]. When choosing the surface treatment method in the bonding process, the type, properties and structure of the joined materials are of great importance [13,14,15,16,17]. Thus, in the bonding process, it is very important to adapt the surface treatment method to the properties of the bonded materials [18,19]. This ensures a joint with appropriate adhesive properties [20,21,22,23]. In order to increase the strength and durability of the adhesive joint, various physical and chemical methods are used, which ensure the removal of contaminants from the joined surfaces, changing the surface structure and increasing the surface free energy, which is related to its good wetting [23,24,25,26,27].

In the bonding process, it is also important to assess the degree of surface treatment, where the phenomenon of adhesion occurs, and the wetting phenomenon. One of the methods used for this is measuring the contact angle and, on the basis on that, determining certain dependencies [16,28]. Prakash and Prasanth [28] emphasized the importance of assessing surface properties by determining, inter alia, the wettability of various processes. They also underlined that the wettability of a liquid on a surface changes with surface properties such as roughness, surface energy or structure. The wetting phenomena on a micro-grooved aluminum surface was presented by Sommers and Jacobi [14]. They underlined that no chemical surface treatment was necessary to achieve water repellency; it was accomplished primarily through the anisotropic surface topography. Depalo and Santomaso [29] presented that the contact angle is an indicator of the liquid–solid affinity and it originates from the equilibrium balance between and cohesive forces. Janssen et al. [30] concluded that contact angle values give a direct indication of solvent wettability and allow the selection of the appropriate solvent or surface treatment to assure a good solvent wetting.

The properties and the surface roughness of adherends materials also have a significant impact [31,32,33,34,35,36,37,38,39]. The importance of creating irregularities was presented in their work by Li et al. [40]. They underlined that the microcraters and the nanopores were obtained after a chemical treatment, the interface in cross-section fit well and formed the interlocks at the microscale, and the polymer resin had a friction interaction at the nanoscale with the metal during de-bonding. Pogorzelski et al. [41] conducted research on quantifying the wettability of metallic (Fe, Al, Cu, brass) surfaces covered with sprayed paints. They noticed that the paint-coated treatment of the originally high surface energy, hydrophilic metallic substrate changed the interfacial force balance due to different interactions. The surface wettability was mainly attributed to the compositional changes at the interface than to the surface roughness. Khaskhoussi et al. [27] also used the surface morphology and contact angle with water to determine the relationship between the surface microstructure and superhydrophobic behavior of coated samples.

Many studies emphasize the importance of the dependence of adhesive properties, including various methods of the surface treatment and the strength of adhesive joints of many construction materials [13,16,28,31,42]. However, due to the variety of materials and methods of the surface treatment in terms of the developing areas of the use of adhesive joints and joints, and various methods of assessing these dependencies, it is still a current research topic.

The conducted research aimed to determine the influence of the surface treatment on the adhesive properties of steel sheet surfaces and the strength of the adhesive joints. The conducted tests were aimed at assessing the degree of the surface treatment of steel sheets in the bonding process. Two variants of the surface treatment were used: without a paint coating and with a paint coating, divided into two groups (without degreasing and with degreasing). Additionally, in the case of the analysis of the steel samples without the paint coating, for half of these samples, mechanical treatment was applied. Two-component epoxy adhesive, prepared on the basis of bisphenol A and a polyamide curing agent, was used to prepare the single-lap adhesive joints of the steel sheets. The experimental tests determined: (i) the adhesive properties of the surface of the steel adherends based on the measurement of the contact angle of polar and apolar liquids (including wettability, work of adhesion and surface free energy), (ii) surface roughness parameters (PN EN ISO 4287), and (iii) mechanical properties of the steel sheets adhesive joints (EN DIN 1465).

## 2. Materials and Methods

### 2.1. Adherend

The adherend samples were prepared using 1.0503 (PN-EN 10027-2) unalloyed carbon steel of higher quality. The mechanical properties of the adherends samples are presented in Table 1. The cuboid-shaped samples had the following dimensions: 2 (±0.01) mm thicknesses, 20 (±0.01) mm widths, and 100 (±0.02) mm lengths.

### 2.2. Adhesive

Two-component epoxy adhesive was used to prepare the single-lap adhesive joints. The epoxy adhesive contained a modified epoxy resin based on bisphenol A with an epoxy number of 0.40 mol/100 g (Epidian 57—trade name, Sarzyna Resins, Nowa Sarzyna, Poland) and polyamide curing agent, an amine number, between 290 and 360 mg KOH/g (PAC—trade name, Sarzyna Resins, Nowa Sarzyna, Poland). The epoxy resin and the amide curing agent were mixed in a stoichiometric ratio of resin and curing agent of 100:80. The properties of individual components of the adhesive and the method of the epoxy adhesive preparation are described in [43].

### 2.3. Surface Treatment Prior to Bonding

Two variants of the surface treatment were used: without a paint coating and with a paint coating, divided into two groups (without mechanical and with mechanical treatment). The list of surface treatment methods is summarized in Table 2.

Mechanical processing was performed manually with P500 sandpaper. Twenty circular movements were made under manual pressure, resulting in a non-directional structure. In order to remove impurities and sediment residues, the samples (A2, B2) were degreased before applying the paint coating. Degreasing consisted of wiping the joined surfaces of the samples with the preparation [44]. Degreasing was performed by spraying with technical acetone (Wach, Janów Lubelski, Poland). The time between the surface treatment of the samples (both mechanical and degreasing) and the application of the adhesive was 1–2 min.

An anti-corrosive metal paint was used to apply the paint coating (JEDYNKA^®^ NEOKOR^®^, Tikkurila, Dębica, Poland). It is a paint based on styrene alkyd resin with the addition of auxiliary and anti-corrosion. Before using the paint, the product was thoroughly mixed with a flat mixer for 2 min. Then, the first layer was applied with a soft flat brush, and the paint was applied once continuously, so as not to disturb the structure of the applied layer. The second layer was applied by the “wet on wet” method. The drying time of the coating in atmospheric conditions (i.e., temperature 21 ± 1 °C and relative air humidity 20 ± 5%) was 8 h. The temperature of the surface to be painted was maintained, which should be at least 3 °C above the dew point temperature.

### 2.4. Adhesive Joints

**Figure 1:**
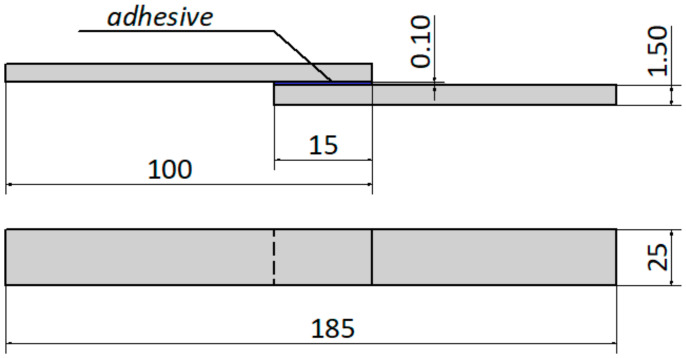
Shape and dimension of single-lap adhesive joints (theoretical dimensions in mm).

Strength tests were carried out on adhesive joints made of C45 steel sheets, overlapped with a lap length l = 15 mm. The shape and dimensions of samples used for strength tests are shown in Figure 1.

A two-component epoxy adhesive was used to make the adhesive joints (described in Section 2.2). The obtained adhesive mixture was applied to the previously prepared surface, in accordance with the adopted overlap length of 15 mm. The epoxy adhesive was applied manually by means of a roller coater in one application on one of the adherend surfaces and fixed in a special fixing device. Due to the use of a special holder (assembly jig for fixing), it was possible to obtain a repeatable thickness of the adhesive joint mentioned above. The preparing adhesive joint samples were performed at a temperature +21 ± 1 °C and relative air humidity 20 ± 5%. In the conducted tests, and a total of 24 joints were made, which were: 12 joints without paint coating (A1—6 adhesive joints, B1—6 adhesive joints) and 12 adhesive joints with paint coating (A2—6 adhesive joints, B2—6 adhesive joints). The created adhesive joints were cured at the ambient temperature (21 ± 1 °C) for 8 days, under a pressure of 0.04 MPa. A special holder (assembly jig for fixing) was used to provide and maintain constant 0.04 MPa. In the case of the epoxy adhesive used, it was possible to use a single-stage cold curing.

The adhesive joints prepared in this way were subjected to measurement control, the results of which were used to perform strength tests, and these, in turn, allowed us to determine the shear strength of the adhesive joints in relation to the variable method of the surface treatment.

### 2.5. Tests

The conducted experimental studies consisted of:

- Determining the adhesive properties of the surfaces of the adherend, based on the measurement of the contact angle, assessing the wettability and the work of adhesion, and determining the value of the surface free energy and its components;
- Determining the surface roughness characteristics by measuring the selected parameters of surface roughness;
- Determining the strength and the load capacity of the adhesive joints.

These tests were performed taking into account the adopted methods of the surface treatment. The presented properties and parameters were used to assess the degree of surface treatment before bonding.

#### 2.5.1. Contact Angle Measurement

Measurements of the contact angle were the basis for the assessment of the adhesive properties, which was achieved by determining the surface wettability, work of adhesion and surface free energy. To evaluate the surface wettability and to calculate the work of adhesion, the measurements of the water contact angle were used, while the determination of the value of the surface free energy was based on the results of the measurement of the contact angle with water and diiodomethane.

The measurement of the contact angle was carried out on the adherend, the surfaces of which were subjected to various treatment methods. The characteristics of the surface treatment methods and the designations of the samples used in the surface wettability tests are presented in Table 3. Additionally, samples without paint coating (A1, B1) were degreased with acetone (A1.1; B1.1). After the application of the degreasing agent, the contact angle of the surfaces was measured again.

Two measuring liquids were used for the measurement: distilled water and diiodomethane (Merck KGaA, Damstadt, Germany).

Measurements of the contact angle were performed on the stand for analyzing the adhesive properties of the solid material surface, the main element of which is the Phoenix 300 goniometer (SEO, Saneop-ro, Republic of Korea) and the Surfaceware 7 software, which enables the obtained results to be recorded.

A direct measurement of the contact angle was used. The volumes of the measuring liquids were 2 μL for water and 1.6 μL for diiodomethane. The measuring liquids were applied with a dispenser from a distance of 20 mm from the sample surface. Automatic measurement of the contact angle was performed immediately after the liquid drop was applied to the analyzed surface. For the measurement of the contact angle of the surface, samples according to Table 3 were used, on which 5 measuring drops were applied at different distances from each other by means of a dispenser.

During the measurements, the right and left contact angles were measured, the results were averaged and the mean value of the contact angle was included in the further analysis. Ten measurements were made for each angle for each drop (5 drops) deposited on the surface of the sample. A total of 100 measurements of the contact angle with distilled water and 100 measurements of the diiodomethane contact angle were made for each sample of the adherend.

#### 2.5.2. Wettability

Assessment of surface wettability (degree of wetting) on the basis of the value of the contact angle with distilled water (polar liquid) was made on the basis of the criteria given in Table 4, prepared on the basis of information contained in the works [24].

#### 2.5.3. Work of Adhesion

The contact angle of a liquid on a surface can be related to the thermodynamic work of adhesion (W_a_) [17,18]. The work of adhesion (Wa) was determined on the basis of measurements of the measuring liquids contact angle (water and diidomethane) and using the Young–Dupré Equation (1) [17,18].

W_a_ = γ_L_ (cosΘ + 1), (1)

where: γ_L_ is the surface tension of a measuring liquid (water) and Θ is the contact angle of measuring liquid. 

#### 2.5.4. Surface Free Energy

The values of surface free energy (SEP) and their components: polar and dispersion for both liquids are presented in Table 5. The adopted values [45,46] are used in the Owens–Wendt method, which states that the surface free energy is the sum of the polar and dispersion components (2) [18,45].

γ_S_ = γ_S_^d^ + γ_S_^p^, (2)

where: γ_S_ is the surface free energy, γ_S_^d^ is the dispersion component of surface free energy and γ_S_^p^ is the polar component of surface free energy.

Water is characterized by a high value of the polar component, while diiodomethane is a dispersion liquid. Selecting this measuring liquid enables the material SEP component values to be determined using the Owens–Wendt method [18,47,48,49].

#### 2.5.5. Surface Roughness Parameters

During the tests, the following parameters of the surface roughness of the surface adherend samples were measured in relation to different surface treatment methods (in accordance with the PN—EN ISO 4287 standard): Rz—the greatest profile height, Ra—arithmetic mean of the profile ordinates, RSm—the average width of the grooves of the profile elements, Rmax—the maximum roughness height, Rp—the height of the highest elevation of the profile, Rpm—the average height of the greatest elevation of the roughness profile, Rv—the depth of the lowest profile cavity, Rsk—the profile asymmetry factor, Rku—the profile slope factor, Rq—the mean square of the profile elevation, and Rt—the total profile height.

The Hommel Tester T1000 contact profilometer (Hommelwerke GmbH, Schwenningen, Germany) was used for the study of surface roughness parameters measurements. The device consists of a measuring head and a parameter evaluation unit, together with a printer connected to the built-in roughness standard. The measuring range of the head is ±80 μm, and the measurement resolution is 0.01 μm.

#### 2.5.6. Strength Test

Shear strength tests of the adhesive joints were carried out on a Zwick/Roell Z150 testing machine (ZwickRoell GmbH&Co. KG, Ulm, Germany). The tests were performed in accordance with DIN EN 1465 and the strength test speed was 5 mm/min. The shear test of the steel sheet adhesive joint was performed at a temperature 20 ± 1 °C and relative air humidity 23 ± 2%. The conditioning time of the adhesive joints samples from the end of the curing process was 24 h, and the conditioning took place in the same conditions as the performance of the strength tests.

## 3. Results

### 3.1. Adhesive Properties

To evaluate the adhesive properties, the measurement of the contact angle (average value, Table 6, Table 7, Table 8 and Table 9) was used, and wettability, work of adhesion and the value of the surface free energy and its components were determined.

#### 3.1.1. Contact Angle

**Figure 2:**
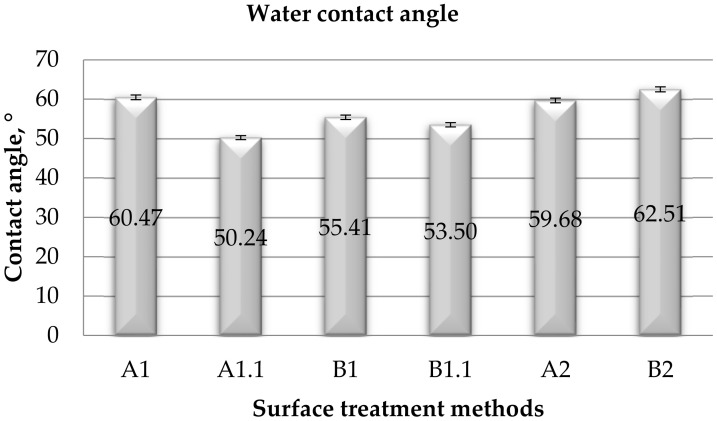
Averaged values of the total (right and left) contact angle with water for individual surface treatment methods.

**Figure 3:**
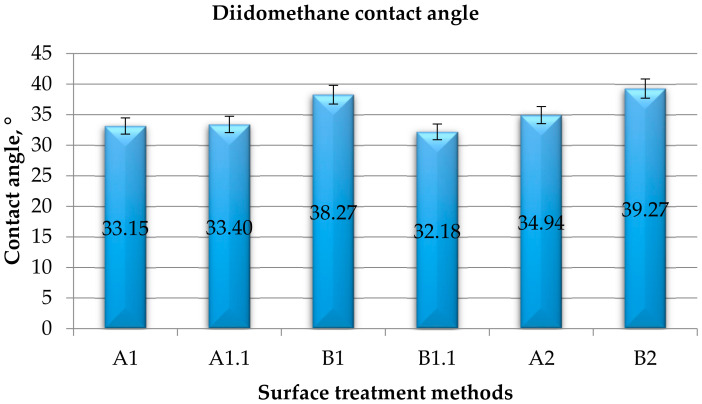
Averaged values of the total (right and left) contact angle with diiodomethane for individual surface treatment methods.

Measurements of the contact angle with distilled water on the surface of steel sheets after various methods of the surface treatment, taking into account the right and left contact angle, are given in Table 6, Table 7, Table 8 and Table 9 present the results obtained for wetting the steel surface with diiodomethane. Water contact angle average value is presented in Figure 2 and diiodomethane contact angle average value is presented in Figure 3.

**Table 6:** The results of the measurements of the right contact angle with water, obtained for a different method of surface treatment.

Right Contact Angle (°)						
Designation of Surface Treatment (Table 3)						
No.	A1	A1.1	B1	B1.1	A2	B2
1	61.95	52.94	62.52	44.98	62.31	55.79
2	67.25	50.89	57.29	52.20	59.85	56.72
3	67.25	50.88	57.77	54.05	60.26	60.06
4	70.15	53.27	54.54	49.65	67.45	60.29
5	61.38	54.08	54.20	54.67	56.37	57.39
6	55.62	52.50	57.53	55.96	53.59	70.61
7	56.19	50.91	51.71	55.00	55.35	70.51
8	57.82	40.48	53.83	54.29	59.64	67.62
9	53.59	46.26	52.26	56.44	61.05	66.83
10	57.05	44.49	55.89	52.15	61.50	58.39
Average	**60.56**	**50.27**	**55.41**	**53.49**	**59.54**	**62.42**
Standard deviation	**4.71**	**3.20**	**2.02**	**2.03**	**2.45**	**5.84**

**Table 7:** The results of the measurements of the left contact angle with water, obtained for a different method of surface treatment.

Left Contact Angle (°)						
Designation of Surface Treatment (Table 3)						
No.	A1	A1.1	B1	B1.1	A2	B2
1	62.27	52.96	62.37	44.64	62.90	55.82
2	66.63	51.20	56.88	52.37	59.86	56.90
3	66.68	50.96	57.89	54.09	60.05	60.33
4	69.99	52.34	54.81	49.52	67.38	60.45
5	61.28	53.97	54.62	54.70	56.12	57.95
6	55.94	52.71	57.31	55.52	54.15	70.78
7	55.73	50.38	51.91	55.00	55.32	70.71
8	57.24	40.63	53.92	54.57	59.46	68.00
9	53.39	46.77	52.12	56.43	62.93	66.58
10	57.15	44.34	55.74	52.20	61.92	58.39
Average	**60.37**	**50.21**	**55.41**	**53.50**	**59.82**	**62.59**
Standard deviation	**4.55**	**3.08**	**1.93**	**2.00**	**2.87**	**5.82**

**Table 8:** The results of the measurements of the right contact angle with diiodomethane, obtained for a different method of surface treatment.

Right Contact Angle (°)						
Designation of Surface Treatment (Table 3)						
No.	A1	A1.1	B1	B1.1	A2	B2
1	28.99	28.02	49.35	32.47	39.33	41.24
2	28.65	31.38	46.94	23.58	38	38.43
3	27.86	37.12	32.61	27.4	29.12	29.45
4	34.69	39.81	28.01	37.55	29.46	39.77
5	29.29	40.61	36.14	28.66	30.65	39.30
6	43.31	29.2	40.97	36.69	34.06	42.03
7	29.37	37.48	39.17	28.21	42.42	46.58
8	36.48	34.54	36.81	32.53	37.52	38.26
9	40.62	29.31	34.02	33.56	35.03	33.97
10	34.18	28.69	39.47	41.88	35.75	42.27
Average	**32.78**	**33.44**	**38.27**	**32.13**	**34.98**	**39.41**
Standard deviation	**4.41**	**4.37**	**4.50**	**3.82**	**3.49**	**2.68**

**Table 9:** The results of the measurements of the left contact angle with diiodomethane, obtained for a different method of surface treatment.

Left Contact Angle (°)						
Designation of Surface Treatment (Table 3)						
No.	A1	A1.1	B1	B1.1	A2	B2
1	27.15	27.94	49.38	32.46	39.75	40.23
2	30.40	30.84	47.14	24.21	37.53	38.43
3	29.22	36.07	32.59	27.46	29.14	29.35
4	35.29	39.54	28.39	37.34	29.21	40.34
5	30.17	40.80	36.18	28.66	30.04	38.70
6	43.81	29.21	41.24	36.88	34.70	41.54
7	30.61	37.82	39.17	28.41	42.17	46.08
8	37.96	34.94	37.07	32.68	37.68	38.54
9	40.15	29.36	33.13	33.95	33.11	34.34
10	34.24	29.13	39.63	41.65	37.10	40.91
Average	**33.51**	**33.36**	**38.27**	**32.23**	**34.89**	**39.13**
Standard deviation	**4.06**	**4.23**	**4.70**	**3.80**	**3.82**	**2.26**

On the basis of the obtained measurement results (Table 6), it can be seen that the highest mean value of the right water contact angle was obtained for the B2 samples and the lowest was obtained for A1.1.

As a result of the direct measurement of the contact angle and the obtained test results, it can be seen that the highest mean value of the left contact angle with water (Table 7) was obtained for the B2 samples. The samples subjected to degreasing have the smallest contact angle, i.e., samples A1.1 and B1.1. By analyzing the results obtained for samples A1 and A1.1, it can be concluded that degreasing the surface caused a significant reduction in the contact angle.

After analyzing the obtained averaged results of the left and right surface contact angles (Table 6 and Table 7), it can be concluded that they differ by a maximum of ±0.19°. Therefore, it can be assumed that the angle is equal in both cases.

On the basis of the obtained results of measurements of the right contact angle of the surface with diiodomethane (Table 8), it can be seen that the highest mean value of the right contact angle was obtained for the B2 samples and the lowest was obtained for B1.1. The performed measurements show that the greatest left contact angle of the surface with diiodomethane (Table 9) was also observed for the B2 samples, and the lowest was observed for the samples with B1.1 surface treatment. As is the case of the left angle, the B1 samples are characterized by an equally large value of the right contact angle. All the above-mentioned samples were treated with P500 sandpaper. The obtained results show that the additionally applied surface degreasing has a significant influence on the change in the contact angle. The contact angle for samples A1 and A1.1 is similar. Applying a coating to these samples increases the value of the diiodomethane contact angle.

After analyzing the obtained averaged results (Figure 3) of the left and right contact angles of surface (Table 8 and Table 9), it can be concluded that the angle is equal in both cases.

**Figure 4:**
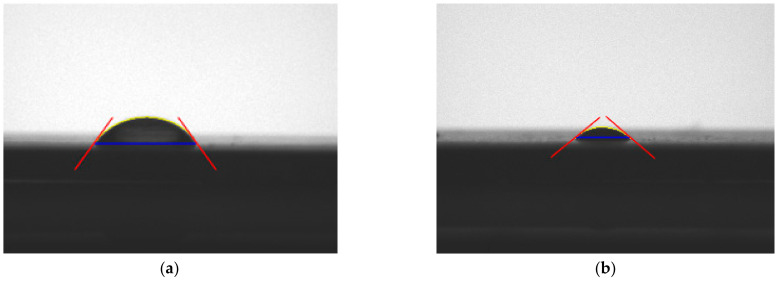
Comparison of the contact angle of the liquids used: (**a**) distilled water, (**b**) diiodomethane.

Figure 4 shows a direct measurement of the contact angle carried out with distilled water and diiodomethane.

#### 3.1.2. Wettability

The wettability of the analyzed surfaces was determined on the basis of the contact angle using distilled water as a polar liquid (Figure 2). A summary of the wettability rating is provided in Table 10 based on the criteria in Table 4.

From the value of the contact angle, it can be seen that a high wettability is obtained after the applied surface treatments (Table 3). The contact angle ranges from 0° to 90°.

#### 3.1.3. Work of Adhesion

**Figure 5:**
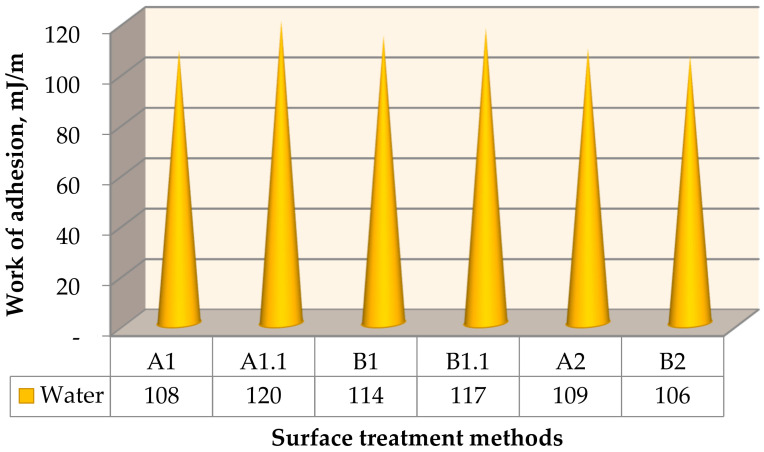
Work of adhesion with water, taking into account different methods of surface treatment.

The averaged results of the work of adhesion measurements for water are shown in Figure 5.

Based on the results obtained (Figure 5), it can be concluded that the highest mean value of the adhesive work was obtained for samples A1.1 and B1.1, and the lowest value was obtained for B2. The samples A1 and A2 were equally valuable. The work of adhesion (Wa) in the case of the variant without surface treatment (A1) for the surface without coatings was comparable to the value of the work of adhesion (Wa) determined for the surface of the samples without surface treatment but with coatings (A2). It can be assumed that, in this case, the presence of the coating does not change the value of the work of adhesion.

#### 3.1.4. Surface Free Energy (SFE)

**Figure 6:**
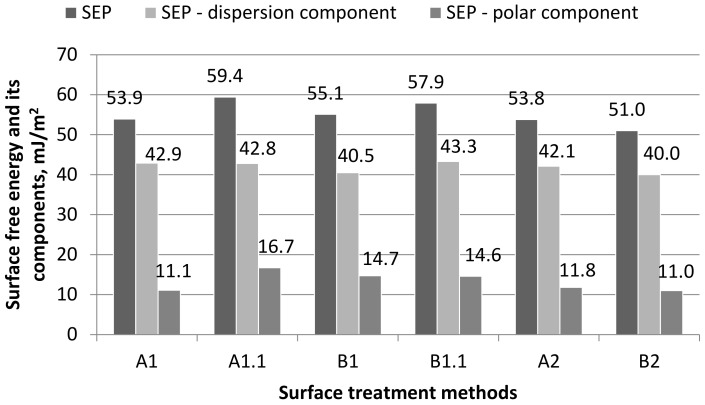
Surface free energy and its components, taking into account different methods of surface treatment.

Based on the measurement of the contact angle with two measuring liquids (Figure 2 and Figure 3) and using the Owens–Wendt method (Section 2.5.4), the value of surface free energy and its components were determined, and the results are presented in Figure 6.

On the basis of the obtained results (Figure 6), it can be concluded that the highest value of surface free energy was obtained for samples A1.1 (59.4 mJ/m^2^)—with only degreasing and B1.1 (57.9 mJ/m^2^)—with mechanical treatment and degreasing, and the lowest value was obtained for B2 (51.0 mJ/m^2^). Samples A1 and A2 were equally valuable in terms of surface free energy. In the case of the dispersion component of SFE, the highest value was obtained for samples B1.1 (43.3 mJ/m^2^) and A1 (42.8 mJ/m^2^). However, in the case of the polar component of SFE, the highest value was obtained for samples A1.1 (16.7 mJ/m^2^) and then for samples B1 (14.7 mJ/m^2^) and B1.1 (14.6 mJ/m^2^), and the lowest value was obtained for samples B2 (11.0 mJ/m^2^) and A1 (11.1 mJ/m^2^). It has been noticed that greater differences in the values of the SFE components occur in the case of the polar component of surface free energy (the difference between the highest and the lowest value is 34%).

### 3.2. Surface Roughness Parameters

**Figure 7:**
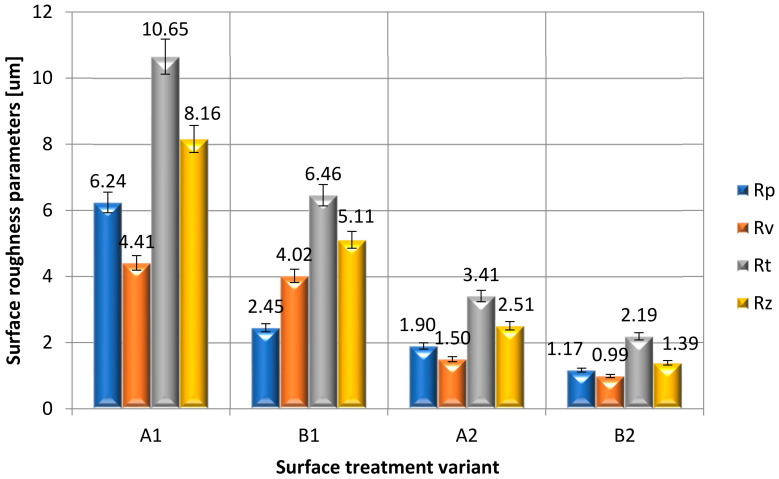
Vertical parameters: Rp, Rv, Rt and Rz of the surface roughness, obtained after applying a different method of surface treatment.

**Figure 8:**
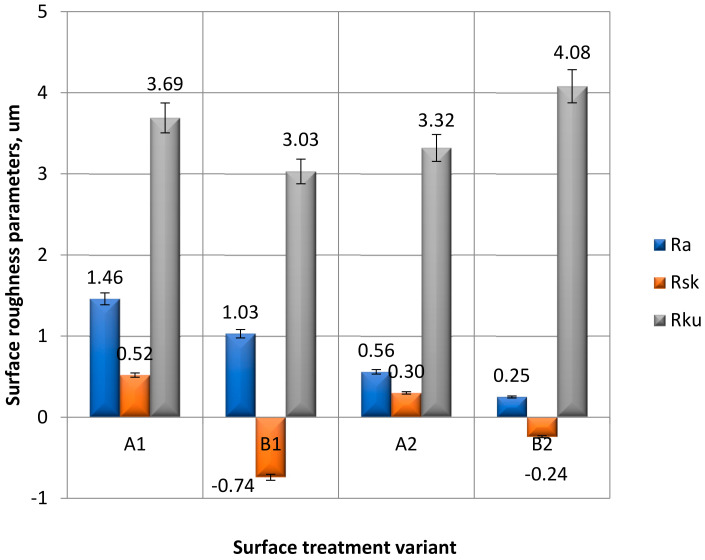
Amplitude parameters: Ra, Rsk and Rku of the surface roughness, obtained after applying a different method of surface treatment.

**Figure 9:**
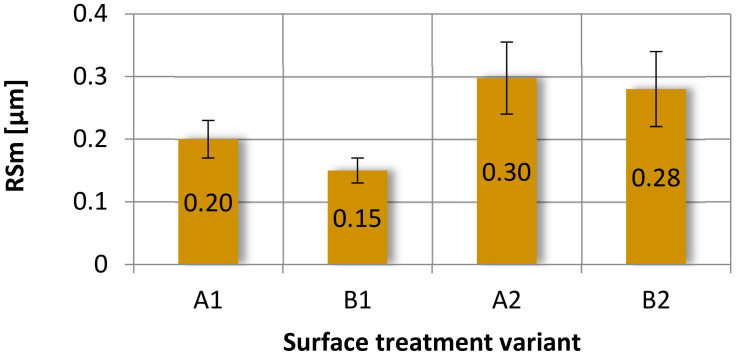
Average width of the grooves of the RSm profile elements, obtained after various surface treatment methods.

The results of the analyzed parameters were divided into three groups:

- Vertical parameters: Rp, Rv, Rt and Rz (Figure 7);
- Amplitude parameters: Ra, Rsk and Rku (Figure 8),
- Horizontal parameter: RSm (Figure 9).

On the basis of the obtained measurement results of the vertical parameters (Figure 7), it can be noticed that the highest value of the high of the surface roughness profile (Rp) was obtained for the A1 samples, and the lowest for the B2 samples. The averaged results of the measurements of the surface roughness parameter Rv of steel samples C45 proved that the value of the depth of the lowest cavity of the Rv surface roughness profile decreases when the surface is covered with a paint coating. The use of sandpaper to prepare the surface (B1, B2) causes an additional decrease in the value of this roughness parameter, both in the case of covering the surface of the samples with a paint coating (A2, B2) and samples without the application of the coating (A1, B1). The averaged results of the surface roughness measurements of the C45 steel samples showed that the value of the surface roughness parameter Rt decreased when the surface is covered with a paint coating and sandpaper is used for surface preparation. The lowest value of the Rt parameter was observed for samples with preparation B2, and the highest was for samples A1. On the basis of the obtained measurement results (Figure 7), it can be seen that the surfaces of A1 and B1 samples were characterized by the Rz greatest height parameter of the surface roughness. Much lower values were observed when the paint coating was applied on the surface of A2 and B2 samples. It can also be concluded that the value of the Rz parameter decreases in the case of using the mechanical treatment to prepare the adherends’ surface.

The performed measurements of amplitude surface roughness parameters (Figure 8) showed that the highest arithmetic mean of surface roughness profile deviations Ra was observed for the surfaces of A1 and B1 samples, and the lowest was observed for A2 and B2 samples (Figure 8). It can be seen that the surface roughness parameter Ra, as is the case with the Rz parameter, decreases with the application of a paint coating and with the preparation of the sample surface by means of mechanical treatment. Analyzing the obtained results of measurements of the asymmetry value of the surface roughness profile Rsk, it can be noticed that for samples with surface preparation using mechanical treatment (B1 and B2), negative values were obtained. It can also be seen that the application of a paint coating reduces the value of this coefficient. A negative value of the asymmetry of the profile indicates that the elevations of the profile have flat peaks, while the recesses have long narrow gaps. The results of the Rku surface roughness profile slope measurements showed that the highest averaged values were obtained for the B2 samples, and the lowest were obtained for the B1 samples. In the case of samples covered with a paint coating (A2, B2), the use of sandpaper before applying the paint coating (B2) increases the value of the Rku parameter, compared to samples with preparation A2. In the case of samples without paint coating (A1, B1), the value of this parameter is at the opposite level, i.e., the Rku parameter decreases for samples prepared by mechanical treatment (B1) compared to samples with preparation A1.

The summary of the average width of the grooves of the RSm profile elements of the tested sample surfaces as a representative of the horizontal parameters is shown in Figure 9. 

Analyzing the obtained results of measurements of the average width of the grooves of the RSm profile elements, it can be seen that the maximum mean value of this parameter was obtained by the samples using the A2 and B2 surface treatment (Figure 9). Samples A1 and B1 showed significantly lower values of the roughness parameter RSm. It can be seen that the application of a paint coating causes an increase in the value of the surface roughness parameter RSm. In addition, the use of mechanical treatment to prepare the surface reduces the value of this parameter.

The profilograms of the surface of steel sheets after various methods of surface preparation are presented in Table 11.

It is noted that the surface of the samples after machining (B1) is characterized by a greater amount of micro-unevenness, while in comparison to the untreated surface (A1), there was a reduction in the height of the surface irregularities and a greater regularity of the irregularities.

### 3.3. Shear Strength

**Figure 10:**
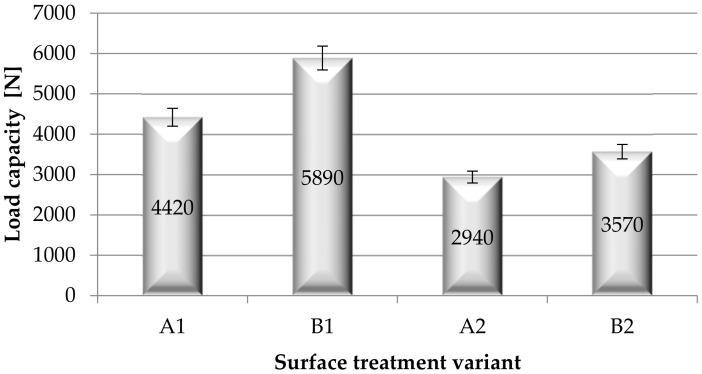
Load capacity of adhesive joints for various methods of surface treatment of the adherend.

**Figure 11:**
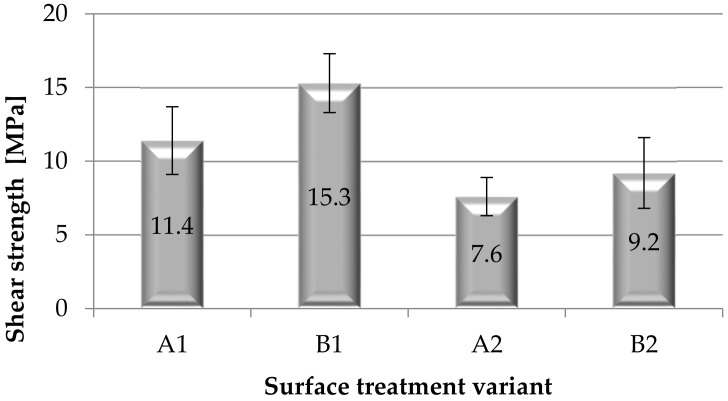
Shear strength of adhesive joints for various methods of surface treatment of the adherend.

Based on the strength tests, the load capacity (Figure 10) and the shear strength (Figure 11) of the adhesive joints of steel sheets were determined, after various surface treatment methods of the steel adherend.

On the basis of the analysis of the obtained results (Figure 10), it can be noticed that the greatest load capacity is transferred by the adhesive joints with the B1 variant (mechanical treatment of surface samples), and the lowest is transferred by the joints with the surface treatment of A2 and B2 (variants with coating). Covering the surface of the samples with a paint coating reduces the load-bearing capacity, and thus proves the low strength of the adhesive joints. When analyzing the obtained results (Figure 11), it can be seen that the adhesive joints obtained as a result of surface treatment B1 have the highest strength, while A2 and B2 have the lowest strength. Thus, it can be concluded that the use of a paint coating adversely affects the adhesive joints strength. The use of B1 surface treatment (mechanical surface treatment) gives the adhesive joints 25.5% higher shear strength compared to the adhesive joints’ strength after A1 surface treatment (adherends without surface treatment). During the visual assessment of the destruction of the samples of adhesive joints (according to EN ISO 10365), no significant differences in the nature of failure were observed, as the failure assessment showed failure in almost all SCF type samples (special cohesive failure). This type of failure was also noticed in the case of the adhesive joints of the elements covered with the paint coating. In this case, no damage to the paint coating was observed—e.g., its separation from the substrate, and failure of the adhesive layer (cohesive failure of the adhesive) was observed.

It should be also noted that properties of adherends do not affect the results because the highest stresses in the steel in the tests are in the order of 150 MPa.

## 4. Discussion

Comparing the obtained measurements of the adhesive properties, there is a noticeable difference between the obtained values of the contact angle, the work of adhesion and surface free energy, resulting from the different surface treatment variants. The measurements of the contact angles of the surface with diiodomethane have significantly higher values of the measurement uncertainties, which proves the greater accuracy of the measurements of surface wetting with distilled water.

### 4.1. Wetting

When wetting the surface with water (Figure 2), the most advantageous method of the surface treatment of the steel adherend was obtained for steel sheet samples subjected to surface treatment with the use of degreasing: A1.1 and B1.1. The samples marked B1 are characterized by a slightly lower value, but are equally high. The difference in the values between samples B1 and B1.1 is due to the relationship with the degreasing of the surface. The lowest values of the adhesion work were observed in the case of samples with B2 surface treatment, i.e., samples treated with sandpaper and covered with a paint coating.

Considering the criteria for assessing surface wettability, it can be noticed that in the case of both measuring liquids, polar–distilled water and apolar–diiodomethane, it is possible to assess the degree of surface wetting after various methods of surface treatment with these liquids as high wettability (Table 5). The contact angle of the polar liquid is in the range of 0° to 90°. It can be seen that as the value of the contact angle of the surface increases, the value of the adhesion work decreases. It was found that the most hydrophilic is the surface where only a degreaser was used for surface treatment (A1.1). On the other hand, the least hydrophilic surface (showing the highest contact angle is the surface of steel that is only mechanically treated (B2)).

According to ASTM D5946, the contact angle in itself is not a completely acceptable measure of ink, coating, or adhesive adhesion, but the values of contact angle can be used to define the level of surface treatment. In the analyzed cases of surface treatment, the value of the contact angle with water is in the range from 50.24° to 62.51°, and, in accordance with the ASTM D5946 standard, these methods of surface treatment can be described as very high treatment, because the contact angle is less than 71°. However, it should be remembered that this standard applies to polymer film surfaces.

It should also be argued that the value of the contact angle is also relevant to the accepted theory of the determination of wettability of surface and the contact angle, which is described in many works, inter alia, Prakash and Prasanth [28], Sommers and Jacobi [13], Baldan [17], Zhang [22]. The basic influence on the surface wettability is caused by surface roughness and topology, which has been emphasized in many works [21,28]. However, according to Wenzel theory, roughness has only partial impact in influencing surface wettability, which was emphasized by Prakash and Prasanth [28]. Shahid and Hashim [21] underlined that the relationship between roughness and adhesion is not very simple and proper surface roughness varies from one adhesive to another, and depends upon the type of stress applied. Therefore, in this work, different adhesives were also analyzed.

Conclusion: The surface of the samples after the applied methods of surface preparation is characterized by good wettability. The most hydrophilic is the surface of the adherends samples, which was subjected only to the degreasing.

### 4.2. Work of Adhesion

It was noticed that the use of degreasing in variants of the surface treatment, both without prior treatment and after the mechanical treatment, influences the achievement of a higher value of the adhesive work compared to the variants of surface treatment without degreasing. According to Packham [50], the contact angle approach gives values of surface energies and of the work of adhesion (Wa) that can be usefully applied in the context of the adhesion theory. Thus, the value of the work of adhesion can also be used to assess the adhesive properties of the surfaces of adherends, or to make other types of the adhesive joints.

Conclusion: Degreasing has a positive effect on the increase in the value of the work of adhesion, considered as one of the thermodynamic quantities describing the phenomenon of adhesion [16,18,50]. Therefore, it is recommended to use the degreasing method for better energy properties.

### 4.3. Surface Free Energy

Relating the obtained results of the surface free energy value to the results of other authors, it is noted that an increase in the value of the surface free energy and its components (especially the polar component) is observed after the application of degreasing (A1.1) compared to the surface without preparation treatment (A1). Moreover, degreasing after mechanical treatment (B1.1) allows comparable values of both the total value of surface free energy and its individual components to be obtained. Machining (B1, B1.1) allows the highest value of surface free energy and the polar component of SEP to be obtained. According to the information provided in the ASTM D5946 standard, the stronger the treatment, the more actively the surface reacts with different polar interfaces. Mantel and Wightman [20] noticed that wettability or surface energy measurements have been performed on the treated surfaces and it was found that the surface energy of the metal decays with the inverse of the contamination layer thickness. As these cleaning treatments lead to different oxide layers, it is concluded that the nature of the oxide layer is not the main parameter that determines the surface free energy of metals.

Conclusion: As in the case of the adhesion phenomenon, degreasing has a positive effect on the increase in the value of surface free energy, which is also one of the measures of the adhesive properties. On this basis, it can be assumed that the use of surface treatment variants with degreasing will contribute to making the adhesive joints significantly stronger, although this is not the only decisive factor.

### 4.4. Surface Roughness Parameters

Based on the obtained results of surface roughness measurements of C45 steel for individual preparation methods, it can be seen that the highest value of surface roughness parameters is shown by samples with A1 surface treatment. These samples also show the real shape of the surface, due to the lack of mechanical treatment and paint coating. The lowest values were obtained for samples covered with a paint coating (A2 and B2). On the basis of the obtained profilograms, it can also be seen that samples A2 and B2 have a similar surface structure. A much different nature of the surface is visible in the case of the B1 samples. The surfaces of these samples are characterized by a large number of micro-inequalities with a high value of the profile asymmetry coefficient, which proves the different dimensions of the surface micro-inequalities. Baldan [16] underlined that the relationship between surface roughness and adhesion phenomenon is a rather complex relationship; it is not unequivocal. Ibrahim et al. [51] pointed out that both the chemical composition and topography of the surface influence the wetting phenomenon is considered.

Conclusion: Mechanical treatment increases the number of micro-inequalities, which, from the point of view of the theory of the mechanical adhesion, are beneficial due to the increase in the actual bonding surface. The adhesive fills in the micro-inequalities and creates mechanical anchors after the curing process, as a result, the adhesive bond is stronger.

Comparing the obtained values of the surface roughness parameters with the obtained values of shear strength of the joints made, it can be stated that the surface roughness measurements will not be related to the method of the surface treatment in the bonding process in every case. Despite the average values of the height parameters of the surface, the B1 samples have the best shear strength, and thus they speak of the most advantageous surface treatment in the bonding process for C45 steel. The structure of these samples, visible on the profilogram, differs significantly from other methods of surface preparation. Therefore, it should be stated that the number and dimensions of the micro-inequalities of the surface of the samples with B1 treatment provide the best mechanical adhesion for C45 steel. In the case of these samples, the application of mechanical treatment creates a large number of cavities that allow the adhesive to penetrate and obtain strong mechanical anchors, and thus prevent failure at the interface of both phases. Ibrahim et al. [51] underlined that the shape of peaks is the most important feature of the surface topography.

An equally high value of the strength of the adhesive joints was obtained for samples A1 (without treatment). Measurements of these samples showed high values of roughness height parameters, which is the result of a lack of surface treatment. This means that the adhesive penetrates equally well into the recesses of the natural surface structure, created after the rolling process, and thus ensures a sufficiently high adhesion of the adhesive to the surfaces to be joined. Moldovan et al. [21] shown that the analysis of surface roughness and wettability is critical for assessing the surface functionality, characteristics and properties of the stainless steel surface after the analyzed process in this article. The values of the surface roughness and the contact angle are directly proportional to the number of repetitions and inversely proportional to the speed of this process. Cereška et al. [52] also demonstrated the possibility of using wettability to assess surfaces after laser treatment, obtaining various texture patterns. Khaskhoussi et al. [27] emphasized that the surface wettability of the aluminum alloy can be modified by selecting the specific roughening treatment in combination with the appropriate one chemical modification of the surface. On the basis of the above information, it can also be concluded that the wettability of the surface can be used as one of the indicators of the degree of the surface treatment for various processes.

## 5. Conclusions

The obtained results led to the conclusion that the method of the surface treatment of adherend surfaces affects the adhesive properties of the surface of steel sheets and the strength of adhesive joints. Assessing the degree of the surface treatment for bonding allowed us to determine the adhesive properties of the surfaces of the adherend based on contact angle values: wettability, adhesion work and surface free energy, and also surface roughness parameters. Based on the results, the following conclusions can be noticed:

- More favorable wetting of the surface with distilled water was obtained for samples with surface preparation with degreasing. In addition, the use of degreasing has a positive effect on increasing the value of the work of adhesion (Wa).
- An increase in the value of the surface free energy and its components (especially the polar component) is observed after the application of degreasing compared to the surface without surface treatment. Moreover, degreasing after mechanical treatment allows comparable values to be obtained of both the total value of the surface free energy and its individual components. Machining allows a high value of the surface free energy and the polar component of the surface free energy to be obtained.
- Despite the average values of the surface height parameters, the samples after machining treatment have better shear strength than in the case of other analyzed surface preparation methods, and thus indicate that the surface treatment method is recommended in the bonding process for C45 steel. The use of the machining treatment creates a large number of recesses, allowing the adhesive to penetrate and create strong anchors, thus obtaining the best mechanical adhesion. Therefore, it can be assumed that the dimensions of the micro-inequalities of the surface of B1 samples, visible on the profilograms, provide the best mechanical adhesion for the C45 steel surface.
- Applied paint coatings for the adhesive joints made of C45 steel adversely affect the strength of these joints.
- The use of paint coatings on the surfaces of C45 steel samples that were subjected to bonding also contributed to the achievement of the lower adhesion work, the lower values of the polar component of the surface free energy and the higher values of the contact angle. It can, therefore, be concluded that, in terms of the adhesive properties, the use of these coatings in the discussed case is not favorable, taking into account the properties mentioned.

Based on the test results, it can be seen that the assessment of the degree of the surface treatment of the steel adherends by determining the adhesive properties may constitute some guidelines for the selection of the method of surface treatment before bonding. It has been noticed that basic surface treatment, including degreasing and mechanical treatment, contribute to obtaining good wettability and favorable energy properties, although the surface roughness parameters should also be taken into account. The issues of the adhesion phenomenon are complex, which has been repeatedly confirmed in many studies and therefore should be considered comprehensively, although the first stage of the surface treatment assessment is undoubtedly very important. The strength of the adhesive joints is a partial verification of the surface treatment stage, although it is only a part of the adhesive technology that affects the final strength of the adhesive joints.

## Figures and Tables

**Table 1:** Mechanical properties of steel adherends.

Properties	Adherend—1.0503 Steel
Elastic modulus E (GPa)	198–207
Tensile strength Rm (MPa)	560–850
Elongation A_5_ (%)	14–17
Yield point Re (MPa)	275–490
Hardness in a softened state (HB)	≤229

**Table 2:** Steel adherends surface treatment variants prior to bonding.

Samples Without Paint Coating		Samples with Paint Coating	
Characteristics	Designation	Characteristics	Designation
Without surface treatment	A1	Without surface treatment	A2
Mechanical treatment	B1	Mechanical treatment	B2

**Table 3:** Surface treatment variants prior to contact angle measurement.

Samples without Paint Coating		Samples with Paint Coating	
Characteristics	Designation	Characteristics	Designation
Without surface treatment	A1	Without surface treatment	A2
Without surface treatment and degreasing	A1.1		
Mechanical treatment	B1	Mechanical treatment	B2
Mechanical treatment and degreasing	B1.1		

**Table 4:** Criteria for assessing surface wettability.

Contact Angle	Degree of Wetting	Interaction Strength	
		Solid–Liquid	Liquid–Liquid
θ = 0°	Perfect wetting	Strong	Weak
0° < θ < 90°	High wettability	Strong	Strong
		Weak	Weak
90° ≤ θ < 180°	Low wettability	Weak	Strong
θ = 180°	Non-wetting	Weak	Strong

**Table 5:** Values of SEP and their individual components for the measuring liquids used in the research and the analyzed material.

Measuring Liquid	Surface Free Energy [mJ/m^2^]	Dispersion Component of SFE [mJ/m^2^]	Polar Component of SFE [mJ/m^2^]
Water (distilled)	72.8	21.8	51.0
Diidomethane	50.8	48.5	2.3

**Table 10:** Surface treatment variants prior to contact angle measurement.

Samples without Paint Coating			Samples with Paint Coating		
Surface Treatment Designation	Contact Angle	Degree of Wetting	Surface Treatment Designation	Contact Angle	Degree of Wetting
A1	60.47°	High wettability	A2	59.68°	High wettability
A1.1	50.24°				
B1	55.41°		B2	62.51°	
B1.1	53.50°				

**Table 11:** Exemplary profilograms of the examined sheet metal surfaces, using various methods of surface treatment.

Surface Treatment Designation	Profilograms ^1^
A1	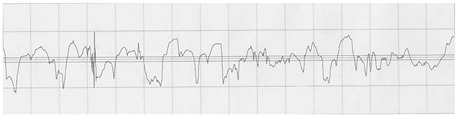
B1	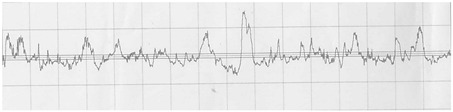
A2	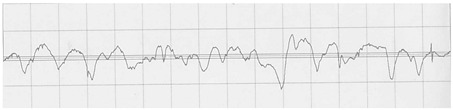
B2	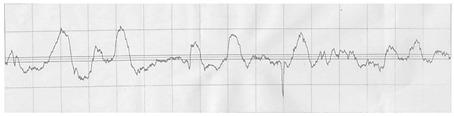

^1^ Measurement conditions: Lt = 4.8 mm, Lc = 0.8 mm, Vt = 0.5 mm/s.

## Data Availability

The raw/processed data required to reproduce these findings cannot be shared at this time due to technical or time limitations.
